# Attentional prioritisation and facilitation for similar stimuli in visual working memory

**DOI:** 10.1007/s00426-023-01790-3

**Published:** 2023-01-12

**Authors:** Zachary Hamblin-Frohman, Jia Xuan Low, Stefanie I. Becker

**Affiliations:** School of Psychology, The University of Queensland, 1/18 Archibald Street, West End, QLD 4101 Australia

## Abstract

Visual working memory (VWM) allows for the brief retention of approximately three to four items. Interestingly, when these items are similar to each other in a feature domain, memory recall performance is elevated compared to when they are dissimilar. This similarity benefit is currently not accounted for by models of VWM. Previous research has suggested that this similarity benefit may arise from selective attentional prioritisation in the maintenance phase. However, the similarity effect has not been contrasted under circumstances where dissimilar item types can adequately compete for memory resources. In Experiment 1, similarity benefits were seen for all-similar over all-dissimilar displays. This was also seen in mixed displays, change detection performance was higher when one of the two similar items changed, compared to when the dissimilar item changed. Surprisingly, the similarity effect was stronger in these mixed displays then when comparing the all-similar and all-dissimilar. Experiment 2 investigated this further by examining how attention was allocated in the memory encoding phase via eye movements. Results revealed that attention prioritised similar over dissimilar items in the mixed displays. Similar items were more likely to receive the first fixation and were fixated more often than dissimilar items. Furthermore, dwell times were elongated for dissimilar items, suggesting that encoding was less efficient. These results suggest that there is an attentional strategy towards prioritising similar items over dissimilar items, and that this strategy’s influence can be observed in the memory encoding phase.

## Introduction

Visual working memory (VWM) is a capacity-limited system that allows for the short-term retention of visual information. VWM is limited in that it can store only three to four visual objects (Cowan, 2001), which can explain our frequent failure to detect changes in a complex visual scene (Rensink et al., 1997). Interestingly, the similarity between visual objects can either facilitate or hinder memory performance. Memory performance is better when objects belong to different categories (e.g., faces and houses) than when they all belong to the same category (e.g., all faces; Cohen et al., 2014; Yang & Mo, 2017), perhaps because items from same semantic categories compete more strongly with each other or are more confusable (Cohen et al., 2014; Franconeri et al., 2013; Wheeler & Treisman, 2002). However, when the memory items all belong to the same category, they can often be recalled better when they are *more* similar to each other (Jiang et al., 2016; Lin & Luck, 2009; Yang & Mo, 2017). In a change detection task, Lin and Luck (2009) found that performance was higher when memory displays contained 3 similar colours (e.g., different hues of green) than when the memory displays contained 3 dissimilar colours (e.g., green, blue, red). This *similarity effect* was also observed in other visual feature domains, including line orientation and length (Sims et al., 2012), unfamiliar characters (Mate & Baqués, 2009), complex faces (Jiang et al., 2016; Yang & Mo, 2017) and landscapes (Yang & Mo, 2017).

It seems plausible that similar items may enjoy an encoding advantage, as similarity could allow encoding of chunks, or relative colours and difference values (rather than only encoding the exact colour values or positions in feature space; Martin & Becker, 2021). Such an encoding advantage would facilitate detecting a change among similar colours (e.g., Jiang et al., 2016; Sims et al., 2012). However, Lin and Luck (2009) argued that the similarity effect is not just due to differences at the encoding stage, as the similarity benefit also emerged for the first presented item in a sequential presentation i.e., before it could be recognised as being part of a ‘similar’ item set (Jiang et al., 2016; Lin & Luck, 2009; Yang & Mo, 2017). Lin and Luck (2009) argued that the similarity effect must therefore reside at the maintenance stage, as an encoding of the first item in a sequence should remain unaffected by the similarity to subsequent items.

Yet, it is still conceivable that the similarity effect may be due to the encoding of relationships or difference values even in sequential presentations, as encoding the second item *in relation* to the first item would re-define the first item even after it has been encoded. That is, encoding the second item as being related to the first item could conceivably restrict the amount of change the first item could undergo without it being noticed, even without changing the way the first item is encoded. Moreover, it seems possible that the first item could be re-coded or encoded with an additional label once the second item is presented, contingent on similarity. To conclude that the similarity effect resides solely in maintenance processes would require separate measurements of how similarity can facilitate the encoding vs. maintenance of memory items.

Another important limitation of previous studies is that the similarity effect was only studied in a very limited range of conditions. Studies have used either simultaneous or sequential presentations of all-similar or all-dissimilar items (e.g., Jiang et al., 2016). Lin and Luck (2009) used a display with a mixture of similar and dissimilar items. However, these were only presented sequentially and thus, without direct competition. Moreover, in the experiment, the probed memory was more likely to be in a similar set than the dissimilar item, which may have provided an incentive to observers to attend more to similar items. Johnson et al. (2009) combined similar and dissimilar items into a mixed display and found that there was a benefit at test for similar items over dissimilar (see also; Peterson et al., 2015; Peterson & Berryhill, 2013). However, these trials were not compared against all-similar or all-dissimilar displays. While similar items were still prioritised in some fashion, it is still unknown if the dissimilar item had some influence on the retention of similar items.

According to current bottom-up attentional selection theories, *mixed* displays should not show a similarity effect, but instead an advantage for dissimilar items (i.e., a reversed similarity effect or dissimilarity effect). This holds because the dissimilar item would be the most visually salient item in the display, and salient items (i.e., items with a high feature contrast) generate an automatic ‘attend-to-me’ signal (Sawaki & Luck, 2010) and thus are prioritized for attention (e.g., Theeuwes, 1992; Wolfe, 1994). As attention is linked to VWM (e.g., Kiyonaga & Egner, 2013; Olivers et al., 2006), prioritized attention to the salient dissimilar item should lead to a prioritisation and hence, interfere with the encoding of the similar items, attenuating the strength of the similarity effect. However, if no attenuation is observed than this would suggest that a dissimilar item did not receive attentional prioritisation.

The aim of the current study was to explore whether the presence of dissimilar items can disrupt or attenuate the similarity effect. In Experiment 1, we found equally large similarity effects for separate and mixed displays, replicating the original similarity effect and extending it to mixed displays where salient (dissimilar) and non-salient, similar items were presented together. Importantly, the similarity effect was found *to the same magnitude* even with favourable conditions for the dissimilar item, which was now the only salient item in the display and equally likely to be tested as the other, non-salient items. In Experiment 2, we additionally recorded eye movements during the encoding stage and revealed that similar items were more likely to be fixated upon than dissimilar, salient items, suggesting that fixations were determined by an attentional strategy prioritising similar items.

## Experiment 1: methods

### Participants

To estimate sample numbers the similarity effect, *F*(1,19) = 15.09, observed in Lin and Luck’s (2009) Experiment 1 was used. To achieve a power of 85% (with 70% assurance) the BUCSS tool suggested a target sample size of 22 (Anderson et al., 2017). Twenty-three volunteers from the University of Queensland, (15 female; *M*_age_ = 21.7 SD = 2.3) participated in this experiment for course credit. All participants reported normal or corrected-to-normal colour vision. Study approval was granted by the University of Queensland’s School of Psychology Ethics Board.

### Apparatus

Stimuli were presented on a 21-in. CRT monitor (refresh: 85 Hz). A chin and headrest held participant’s heads in a constant position 600 mm from the screen. Stimuli were generated via PsychoPy using Python (Peirce, 2007).

### Stimuli

Stimuli were presented against a white backdrop (RGB: [0, 0, 0]), with a black fixation cross (visual angle height: 0.29°) in the centre of the screen. Both memory and test arrays consisted of three coloured squares (1.72° × 1.72°). These squares were positioned at the 9, 12, and 3 o’clock positions of an imaginary circle with a radius of 4.3° from fixation.

**Fig. 1:**
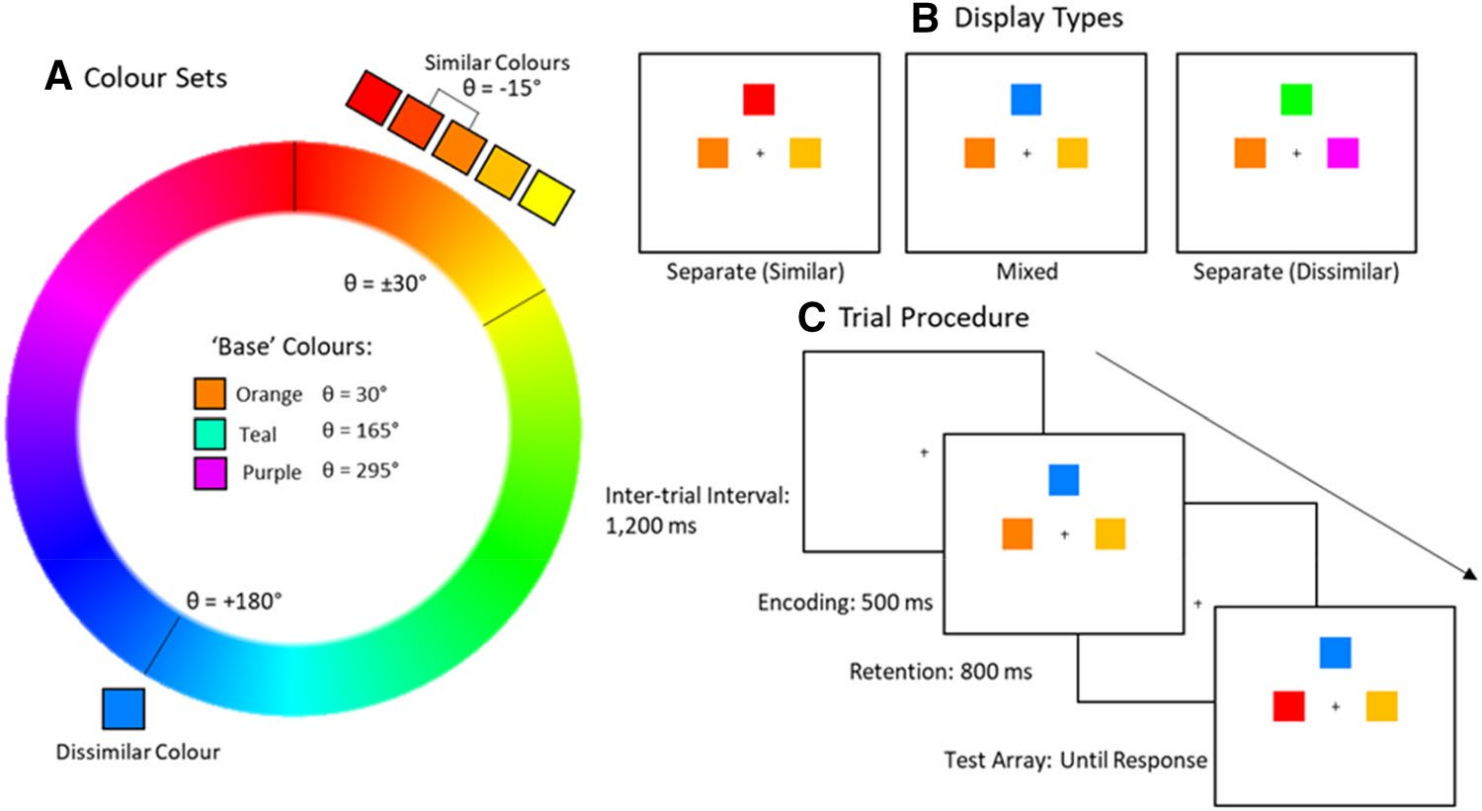
**A** On each trial, similar colours were chosen from one of three sets of five colours centred on orange, teal and purple hues. In each set of similar colours there were five potential hues ± 15 and ± 30° from the base colour. **B** Three types of memory displays were used, defined by the combination of included colours. The ‘all-similar’ display contained hues from the similar set, the ‘all-dissimilar’ display had colours that varied 120° from each other and the ‘mixed’ display contained two similar items and a 180° shifted dissimilar item. **C** Participants had 500 ms to encode the memory items (1200 ms in Experiment 2). After an 800 ms blank screen the memory items returned. On 50% of trials one of the hues had shifted by 30° and on the other 50% trials there was no change. In the mixed displays, the change item had a 50% likelihood to be one of the similar items and 50% likelihood of being the dissimilar item

Three sets of similar colours were created by selecting three base colours from an RGB circle positioned at 30°, 165° and 295° and picking colours located ± 15 and ± 30 degrees of the base colours to form the five similar colours for each set (see Fig. 1). In *all-similar* displays, hues for the three memory items were chosen from these five similar colours. In *all-dissimilar* displays, the three memory colours were selected by using the base colour, plus two other colours that were 120° away from each other and from the base colour. In *mixed displays*, two colours were chosen from the similar set (as in the all-similar displays), and the third, dissimilar colour was created from the opposing side of the colour wheel (180° away from the base colour).

### Design

The experiment consisted of three blocked conditions for a total of 592 experimental trials. In the two separate display conditions, the stimuli were either all similar or all dissimilar (148 trials each). In the other, mixed display conditions (296 trials), two similar items were always presented together with a dissimilar item.

On change trials (50% of all trials), one memory item changed hue by 30°. For mixed displays, changes on a similar and dissimilar item were equiprobable (50% of change trials). This led to the dissimilar item having a proportionally higher chance of changing than an individual similar item, i.e., of the 148 change trials for the mixed displays, 74 were a similar item changing (37 for each item) and 74 were for the single dissimilar item. There was no colour change on the other 50% of trials. Participants completed 16 practice trials with feedback that were not analysed.

### Procedure

Stimuli were displayed for 500 ms in the encoding phase (memory display). After an 800 ms retention interval the test display was presented. The test display could either be identical to the memory display (50% of trials), or one of the items in the display was changed to a hue within the same colour category (i.e., to a similar hue; 50% of trials). Participants were instructed to respond with the ‘s’ key if they believed that all colours had remained the same or with the ‘d’ key if one of the colours had changed. The test screen was displayed until a response was recorded.

## Experiment 1: results

Trials with responses longer than 5000 ms were excluded from analysis (1.0% of trials). Raw errors for each condition were transformed into d’ values to examine change detection sensitivity (Abdi, 2007). Raw accuracy data for trials are reported in the Appendix.

**Fig. 2:**
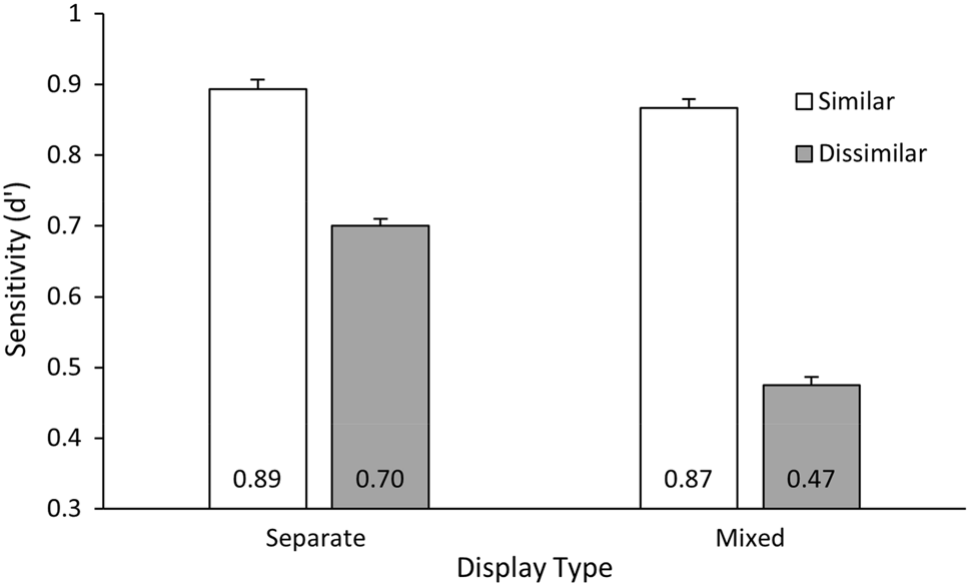
Change detection sensitivity in Experiment 1. Participants were more likely to detect change for similar items over dissimilar, and this effect was more pronounced in the mixed displays than the separate ones. Error bars represent 95% within-subjects confidence intervals (Loftus & Masson, 1994)

A 2 (Change Item: Similar, Dissimilar) × 2 (Display Type: Separate, Mixed) repeated measures ANOVA was conducted on the *d*′ values for the change detection task. Results revealed a main effect of change item, *F*(1, 22) = 1642.95, *p* < 0.001, $\eta_{{\text{p}}}^{2}$ = 0.99, such that change detection was more sensitive for similar items than dissimilar items (see Fig. 2). The effect of display type was also significant *F*(1, 22) = 348.10, *p* < 0.001, $\eta_{{\text{p}}}^{2}$ = 0.94 (reflecting higher sensitivity in the separate over the mixed displays), but importantly this was qualified by a significant interaction, *F*(1, 22) = 376.10, *p* < 0.001, $\eta_{{\text{p}}}^{2}$ = 0.95. Contrary to expectations, the advantage for similar items (*d*′_similar_ − *d*′_dissimilar_) was stronger in the mixed displays than the separate displays (i.e., compared to the difference between the all-similar and all-dissimilar displays), *t*(22) = 19.39, *p* < 0.001, BF_10_ = 2.13 × 10^12^. As shown in Fig. 2, the interaction was due to change detection performance decreasing in the mixed displays compared to the all-dissimilar displays, *t*(22) = 30.55, *p* < 0.001, BF_10_ = 2.05 × 10^16^.

## Experiment 1: discussion

The results of Experiment 1 reproduced the previously observed visual similarity effect (e.g., Lin & Luck, 2009). Change detection sensitivity was higher for the all-similar display compared to the all-dissimilar displays. Importantly, this similarity effect also occurred in the mixed display. Even though the dissimilar item was both more salient and proportionately more likely to be tested than an individual similar item, change detection sensitivity was notably lower. Contrary to our expectations, the similarity benefit was not attenuated in the mixed displays, but instead substantially stronger when the similar and dissimilar items were presented simultaneously. This effect was mainly due to a decrease in memory sensitivity for when the dissimilar item changed.

Contrary to the bottom-up saliency view (e.g., Theeuwes, 1992; Wolfe, 1994), that the salient dissimilar item would be granted prioritised access to VWM, instead the similar items appeared to receive a beneficial allocation of VWM resources. This would suggest a top-down attentional prioritisation or a strategy for encoding similar items that is overriding bottom-up saliency signals to ensure an enhanced representation of similar items in VWM (e.g., Dube et al., 2017; Emrich et al., 2017).

However, it should be noted that this explanation is still speculative, as it is possible that the advantage for similar items arose at a later stage of the change detection task, as for instance, during the maintenance or recall phase of the task (e.g., Lin & Luck, 2009). As Experiment 1 did not use measures that can distinguish between different phases of the task (encoding, maintenance, recall, response execution), it is unknown whether the similarity effect is indeed due to preferential encoding of similar items, only that the supposed attentional prioritisation of salient items did not translate to an advantage for remembering dissimilar items. Instead, the opposite result was observed; a significant detriment for remembering dissimilar items, especially when they were competing with other similar items. Experiment 2 was designed to test the attentional prioritisation explanation, by examining eye movements during the encoding phase of the change detection task.

## Experiment 2: methods

### Participants

Twenty-two volunteers from the University of Queensland, (18 female; *M*_age_ = 22.3 SD = 3.1) participated in this experiment for course credit. All participants reported normal or corrected to normal colour vision.

### Apparatus/stimuli/design/procedure

Eye movements were measured with an SR-Research Eyelink-1000 eye tracker at a 500 Hz sampling rate.

Experiment 2 used identical stimuli as Experiment 1. The only change was that the memory stimuli were now presented 6.4° away from fixation. This change was implemented to encourage overt eye-movements to the stimuli. Furthermore, encoding time was increased to 1200 ms to allow time for multiple fixations. Participants were required to maintain fixation for 500 ms before the trial commenced to ensure gaze started at a neutral position. No other instructions were given to participants regarding eye-movements. Stimulus fixations were recorded as any time gaze was fixated within 2° of the memory stimuli. To avoid large increases in testing time, the number of total trials was reduced to 368 trials, keeping the same proportions of trial types as Experiment 1. This included 184 mixed displays (46 similar item change, 46 dissimilar change and 92 no-change) and 184 separated displays (92 all-similar and 92 all-dissimilar, with 50% change trials).

## Experiment 2: results

### Change detection accuracy

**Fig. 3:**
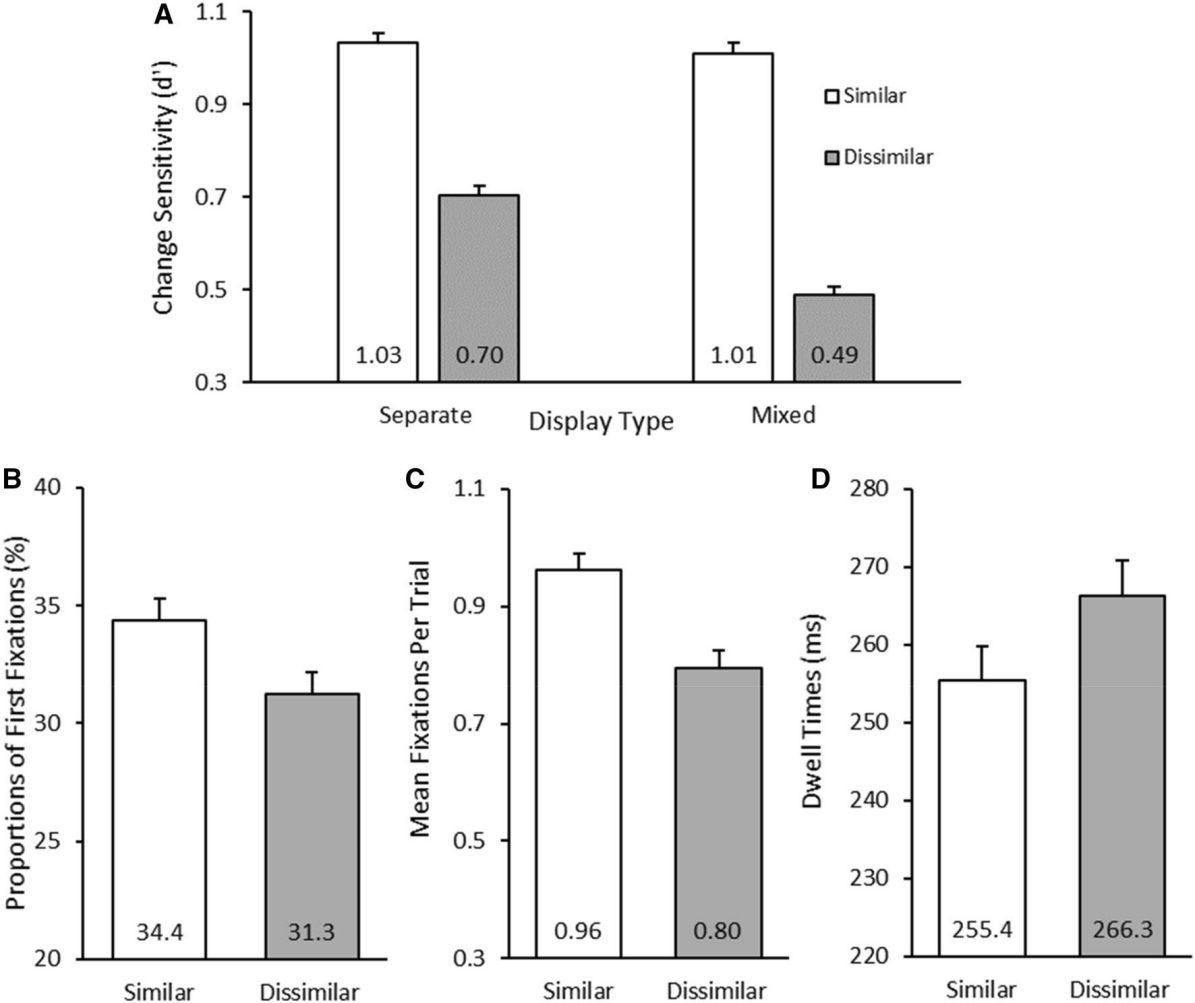
**A** Change detection results for Experiment 2. Participants were more likely to detect changes in similar items than dissimilar items, both in separate and mixed display conditions. **B** In the encoding phase, the first fixation of the trial was more likely to be directed towards either of the similar items than the dissimilar item. **C** The number of fixations during the entire encoding phase showed more overall fixations on similar than dissimilar items. **D** The dwell time results, however, showed that dissimilar items were fixated for a longer duration than similar items. Error bars represent 95% within-subject confidence intervals (Loftus & Masson, 1994)

Memory responses with RTs longer than 5000 ms were excluded from the accuracy analysis (< 1% of trials). A 2 (Change Item: Similar, Dissimilar) × 2 (Display Type: Separate, Mixed) repeated measures ANOVA was conducted on the d’ values for the change detection task. Results revealed a main effect of change item, *F*(1, 21) = 326.61, *p* < 0.001, $\eta_{{\text{p}}}^{2}$ = 0.94, reflecting that change detection performance was higher for similar items than dissimilar items (see Fig. 3a). The effect of display type was also significant *F*(1, 21) = 75.45, *p* < 0.001, $\eta_{{\text{p}}}^{2}$ = 0.78 (reflecting higher sensitivity in the separate over the mixed displays), but this effect was qualified by a significant interaction, *F*(1, 21) = 53.86, *p* < 0.001, $\eta_{{\text{p}}}^{2}$ = 0.72. The similarity effect was stronger for the mixed displays compared to the difference between the separate all-similar and all-dissimilar displays, *t*(21) = 7.34, *p* < 0.001, BF_10_ = 5.13 × 10^5^. The interaction was again due to a decrease in performance for dissimilar items in the mixed display compare to the all-dissimilar display, *t*(21) = 13.38, *p* < 0.001, BF_10_ = 8.64 × 10^8^.

### Gaze analysis

For the analysis of eye-movement data, we excluded trials in which the gaze did not leave the fixation area (17.6% of trials). One participant was excluded from the analysis as they maintained fixation on more than 80% of trials. To gauge whether attention was more strongly attracted to similar or dissimilar items we analysed which type of item received the first eye movement in the mixed displays. As there were two similar items, the probability of fixating on each item was computed as the average proportion of fixations on the two similar items vs. the proportion of fixations on the dissimilar item. The results showed that first eye-movements were more likely to land on either similar item compared to the dissimilar item, *t*(20) = 3.59, *p* = 0.002, BF_10_ = 21.18 (see Fig. 3b).

To assess whether this prioritisation of similar items held for the entire encoding phase in mixed displays, we also analysed the number of fixations on similar vs. dissimilar items (again, using the average of the two similar items to compare to fixations on the dissimilar item). On average there were 2.72 fixations during the encoding phase of the trial. As shown in Fig. 3c, similar items were fixated more frequently than dissimilar item over the course of the encoding phase, *t*(20) = 5.94, *p* < 0.001, BF_10_ = 2623.84.^1^

To assess whether there were any differences in the time needed to encode similar vs. dissimilar items, we compared the mean dwell times on similar (averaged) vs. dissimilar items (i.e., the duration the gaze continuously dwelt within each fixated item). Surprisingly, the mean dwell times were 11 ms longer for dissimilar items compared to similar items in mixed displays, *t*(20) = 2.56, *p* = 0.019, BF_10_ = 3.02 (see Fig. 3d), suggesting that it took more time to encode the colour of the dissimilar item. To see if this effect could also be observed in the separate displays, we also compared the dwell times across the all-similar and all-dissimilar conditions, and again found 11 ms longer dwell times in the all-dissimilar displays (*M* = 264.9 ms) than the all-similar displays (*M* = 254.0 ms), *t*(20) = 2.28, *p* = 0.034, BF_10_ = 1.87.

## Experiment 2: discussion

The behavioural memory accuracy results replicated those from Experiment 1. Change detection sensitivity was greater for similar items than dissimilar in both the separate and mixed displays, and the additional performance cost for dissimilar items in the mixed display was replicated. First eye movements were more likely to land on one of the similar items than the dissimilar ones in the mixed displays, and similar items were likely to receive more fixations overall. Thus, in line with our attentional prioritisation explanation and contrary to bottom-up saliency views, similar items attracted attention and the gaze more strongly than the dissimilar item and this prioritisation lasted for the entire encoding phase, not just the initial attentional allocation.

## General discussion

The results of the two experiments allow novel insights into the visual similarity effect. In Experiment 1 we found that the similarity benefit was robust against interference from a simultaneously presented dissimilar item. Contrary to the saliency encoding hypothesis there was no evidence for the dissimilar item receiving preferential encoding. In fact, change detection sensitivity for the dissimilar item significantly *decreased* in the mixed displays compared to when all items were dissimilar. Even though the dissimilar item was the mostly likely item to be tested in the display, similar items were prioritised in encoding and remembered with higher accuracy. This is corroborated by the eye-movement analysis in Experiment 2, which revealed that similar items were more likely to be fixated first, and were fixated more often in the encoding phase.

Lin and Luck (2009) speculated that the similarity effect may be due to beneficial processes for similar items in the memory maintenance phase. Here we show that the advantage for similar items can also be initiated pre-attentively, as participants seem to have a top-down bias to preferentially select and encode similar items (even with the first eye movement). This attentional bias towards similar items overpowered the bottom-up tendency to preferentially select the most salient item in the display and led to prioritised encoding of similar items in VWM. Conversely, this early attentional benefit in the encoding displays may have been influenced by a stimulus-driven mechanism that overruled salience, perceptual grouping. Similar stimuli can be automatically grouped and encoded, even contradicting top-down goals (Jiang et al., 2004; Shomstein et al., 2010). Potentially the similar items used were grouped and prioritised and then emitted a higher priority signal than the salient dissimilar item. Whether the similarity-encoding benefit is due to a stimulus or goal-driven mechanism is an open question.

It should be noted that the observed bias cannot explain *all* of the similarity effect, as change detection sensitivity for similar items was also higher in the separate display condition, in which the displays contained either all-similar items or all-dissimilar items (and attentional biases are unlikely to play a role). Still, the data show some indication of facilitated encoding of similar items, which may explain the similarity effect. The dwell times results (for both separate and mixed displays) showed that encoding dissimilar items takes more time, reflecting a higher level of difficulty of encoding dissimilar items.

We can currently only speculate about the exact source of this difficulty. However, it is possible that it is easier to encode items in relation to each other rather than by their exact feature value or position in the feature space (Martin & Becker, 2021). For instance, it is imaginable that each encoded colour in the feature space acts like an ‘anchor’ against which the other colours are compared during encoding and maintenance, whereby this comparison process is easier or only feasible for similar items. In addition, the test displays containing all items rather than, for example, a single probe, may make it easier to notice a change in a similar item, as this changes the relations and anchor distances between the items. The latter ‘probe’ effect is, however, unlikely to account for the entire similarity effect, as we found strong and reliable similarity effects already in the encoding stage, and similarity effects have also been reported with single-item probes at the memory test stage (Lin & Luck, 2009).

Both Jiang et al. (2016) and Lin and Luck (2009) speculated that similarity benefits may have been due to a focus on a single similarity dimension. By focussing on this single dimension (e.g., various hues of orange), memory resources can be concentrated in a more efficient way, increasing memory precision. The current results are in line with this proposal, and additionally show an attentional bias for similar colours. In the mixed displays, the similar items were prioritised, perhaps because they formed part of a single similarity dimension. This focus on a specific region of colour space (and/or the corresponding items) could lead to detriments for other regions. This in turn would lead to less precise memory representations for dissimilar items, accounting for the decrease in dissimilar memory accuracy in the mixed displays, compared to the all-dissimilar displays. Furthermore, less efficient processing of dissimilar items could account for the observed increase in dissimilar item dwell times, as it could require more time to encode stimuli from a different colour region. While this explanation is still tenuous, the results of our study show that the similarity effect is a robust and large effect that does not only survive presenting similar and dissimilar stimuli together in the same display but even becomes stronger in these mixed displays. Contrary to previous explanations of the effect (e.g., Lin & Luck, 2009), our results show a clear bias towards similar stimuli originating in the memory encoding phase. Yet, as described by Jiang et al. (2016), the similarity benefit is likely comprised of effects at multiple stages of processing, and further research is required to map out the effect across the different stages of processing and identify the processes and mechanisms responsible for the similarity effect.

## Data Availability

Data, raw and with condition means, can be accessed at https://osf.io/xrn83.

## Appendix

### Raw memory accuracy results

The following are the raw memory accuracy data for Experiments 1 and 2. The differences in d’ reported in the main body of text appear to be driven by detection misses in the change trials and not fluctuations in the false alarm rate (Tables 1 and 2).

**Table 1:** Memory accuracy experiment 1

	All similar (%)	All dissimilar (%)	Mixed (%)
No-change trials	90.2	91.9	91.9
Change trials			
Similar change	78.6	–	76.9
Dissimilar change	–	61.7	45.3

**Table 2:** Memory accuracy experiment 2

	All similar (%)	All dissimilar (%)	Mixed (%)
No-change trials	92.4	93.1	93.4
Change trials			
Similar change	79.0	–	80.4
Dissimilar change	–	61.7	48.5

## Footnotes

1 This analysis was run again, now excluding the first eye movement of the trial. Overall similar items still received more fixations (*M* = 0.62) than the dissimilar item (*M* = 0.48), *t*(20) = 5.29, *p* < .001, BF_10_ = 694.8. This confirms that the similarity effect in the total fixations was not completely driven by the first fixations favouring similar items, but more longer-lasting.
